# Highly Porous 3D Printed Tantalum Scaffolds Have Better Biomechanical and Microstructural Properties than Titanium Scaffolds

**DOI:** 10.1155/2021/2899043

**Published:** 2021-09-28

**Authors:** Huaquan Fan, Shu Deng, Wentao Tang, Aikeremujiang Muheremu, Xianzhe Wu, Peng He, Caihua Tan, Guohua Wang, Jianzhong Tang, Kaixuan Guo, Liu Yang, Fuyou Wang

**Affiliations:** ^1^Center for Joint Surgery, Southwest Hospital, Third Military Medical University, No. 29 Gaotanyan Street, Shapingba District, Chongqing 400038, China; ^2^Chongqing ITMDC Technology Co., Ltd., No. 2, Yangliu Road, Huangshan Avenue, North New District, Chongqing 400038, China; ^3^Department of Spine Surgery, Sixth Affiliated Hospital of Xinjiang Medical University, 39Wuxing Nan Rd, Tianshan District, Urumqi, Xinjiang 86830001, China; ^4^Chongqing Institute of Optics and Mechanics, NO. 2, Yangliu Road, Yubei District, Chongqing 400038, China; ^5^Hunan Printing Medical Instrument CO., Ltd, No. 423 Shuyuan Street, Tianxin District, Changsha 410000, China

## Abstract

**Objective:**

To test the biomechanical properties of 3D printed tantalum and titanium porous scaffolds.

**Methods:**

Four types of tantalum and titanium scaffolds with four alternative pore diameters, #1 (1000-700 *μ*m), #2 (700-1000 *μ*m), #3 (500-800 *μ*m), and #4 (800-500 *μ*m), were molded by selective laser melting technique, and the scaffolds were tested by scanning electronic microscope, uniaxial-compression tests, and Young's modulus tests; they were compared with same size pig femoral bone scaffolds.

**Results:**

Under uniaxial-compression tests, equivalent stress of tantalum scaffold was 411 ± 1.43 MPa, which was significantly larger than the titanium scaffolds (*P* < 0.05). Young's modulus of tantalum scaffold was 2.61 ± 0.02 GPa, which was only half of that of titanium scaffold. The stress-strain curves of tantalum scaffolds were more similar to pig bone scaffolds than titanium scaffolds.

**Conclusion:**

3D printed tantalum scaffolds with varying pore diameters are more similar to actual bone scaffolds compared with titanium scaffolds in biomechanical properties.

## 1. Background

Due to excellent corrosion resistance, toughness, and bioactivity, tantalum has been used for a variety of medical implant since 1940 [1–5]. While titanium is still considered the gold standard for porous biomaterials with skeletal biocompatibility [6–8], tantalum has been increasingly used as bone-substitute material with great potential. Highly porous tantalum scaffold was proven to have good bone conduction and induction capabilities and was shown to integrate well with bone in both basic research and human trials, indicating great prospect in its clinical application [9–11]. However, due to challenges in the processing of tantalum, its application is still limited in musculoskeletal system.

For tantalum scaffolds to have fine osteogenic properties and be easily integrated with the host bone to prevent stress shielding and implant loosening, it is essential to improve the porosity of tantalum scaffold structure to obtain bone like mechanical properties. Previous studies have reported that pore diameter and porosity of tantalum scaffold have significant influence on its biocompatibility and adequate pore diameter, and high porosity is beneficial to the ingrowth of bone, soft tissues, and blood vessels [12–15].

Traditional additive manufacturing techniques such as metal fiber sintering, powder metallurgy, and plasma spraying have been widely used in the production of porous metals. However, metal fiber sintering cannot precisely control the pore parameters [16–18], and traditional powder metallurgy and plasma spraying cannot guarantee the structural uniformity of porous implants [19, 20]. In the meanwhile, newly developed 3D printing technologies made independently controlling pore parameters possible [21]. The first additive manufacturing (AM) processed tantalum structure used laser engineered net shaping technique to create porous tantalum structures with different porosity and tested their in vitro biocompatibility with osteoblast cell lines and MMT assays. The results showed better cell survival, adherence, and extracellular matrix formation with tantalum scaffolds than titanium scaffolds. Considering the high cost of all tantalum implants, Balla et al. used laser engineering net shaping method to deposit tantalum coating on titanium and tested its biocompatibility by osteoblast cell line, which showed significantly better cell adherence and extracellular matrix formation on tantalum coating than titanium scaffolds, further proving the superior cell-material interaction of tantalum.

Although 3D printing appears to be perfect for fine molding of tantalum scaffolds, the methods for designing and assessing the properties of 3D printed tantalum scaffolds are rudimentary. To provide reference for future clinical application of 3D printed tantalum scaffolds, here we modified the existing tantalum scaffold to apply to specific biological sites and tested their structural properties [22].

## 2. Materials and Methods

**Figure 1:**
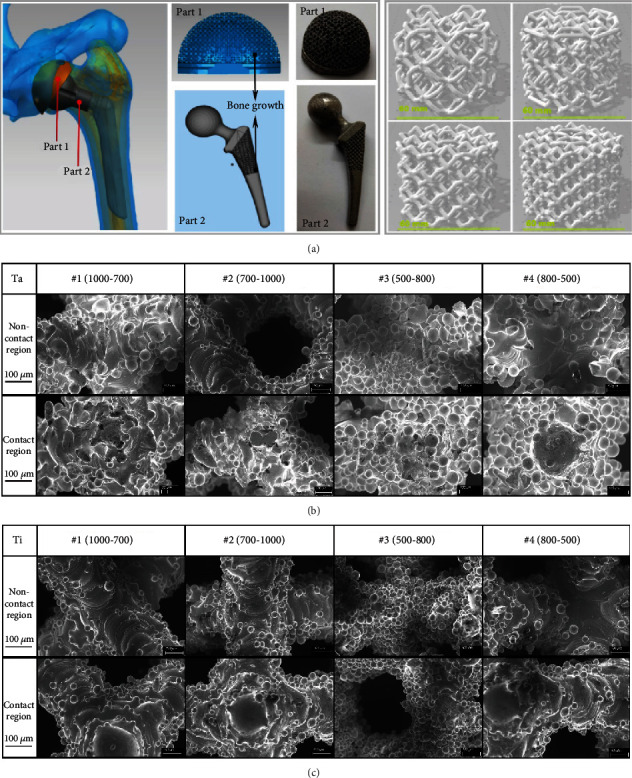
(a) 4 kinds of 3D modeling scaffold and corresponding biological site. (b) SEM images of 4 kinds of tantalum scaffold before compression. (c) SEM images of 4 kinds of titanium scaffold before compression.

**Figure 2:**
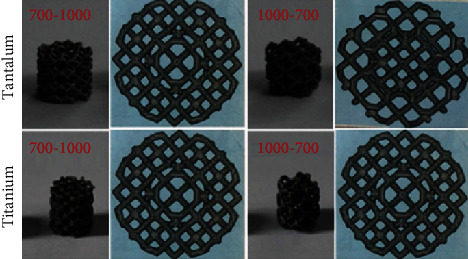
The general image and the cross section of the tantalum and titanium scaffolds.

In the current study, the 3D Max software was used to conduct 3D modeling of the femoral head and acetabulum cup; 4 types of scaffolds with different pore diameter and porosity (Figure 1(a)) were used to replace bone growth part on the implant. Selective laser melting (SLM) was used to mold four types of tantalum and titanium scaffolds with alternative pore diameters: 1000-700 *μ*m (indicating that the inner diameter of the scaffold is 1000 *μ*m, and the outer diameter is 700 *μ*m, Figure 2), 700-1000 *μ*m, 500-800 *μ*m, and 800-500 *μ*m, in contrast to single pore diameter design by previous authors due to molding limitations [23, 24]. The shape of each scaffold was a cylinder with the diameter of 6 mm and the height of 6.8 mm. The corresponding scanning electron microscope (SEM) images are shown in Figures 1(b) and 1(c), respectively.

ZB-YSJ5000 compressive strength tester was used to carry out uniaxial-compression tests on above scaffolds. Compression resistance and fracture of tantalum scaffolds were compared with of titanium scaffolds. In the uniaxial-compression tests, the contact area between scaffold and pressure monitor was named as contact region, and the internal material of scaffold was named as noncontact region. Figures 1(b) and 1(c) show that the scaffolds had no obvious initial cracks either on the surface contact region or in the internal noncontact region.

In order to compare the compression deformation resistance of the above tantalum and titanium scaffolds with that of animal bone, similar biomechanical tests were carried out on five pig proximal femoral bone grafts with the same size as the tantalum and titanium scaffolds. The average test results were used to form the final stress-strain curve.

## 3. Results

**Figure 3:**
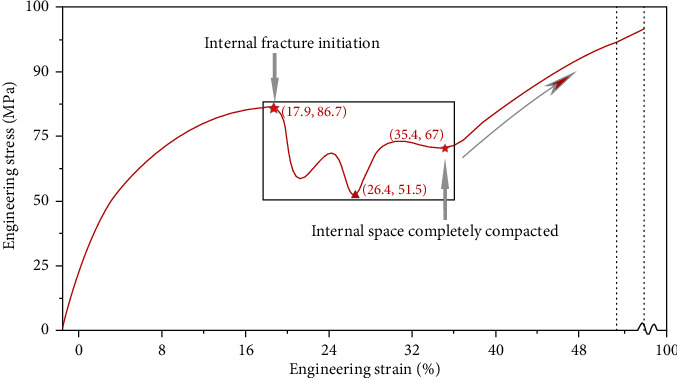
Engineering stress-strain curve of scaffold titanium #2 (700-1000 *μ*m) under the compressive speed of 0.05 mm/s.

The equivalent stress of four types of scaffolds was all significantly larger in tantalum scaffolds than titanium scaffolds (*P* < 0.01, Table 1). The range of Young's modulus of tantalum was 2.61 ± 0.02 GPa-3.03 ± 0.04 GPa, and the range of Young's modulus of titanium was 4.66 ± 0.04 GPa-4.93 ± 0.04 GPa, which was significantly different between the two groups (*P* < 0.01). The engineering stress-strain curve of titanium scaffold #2 (700-1000 *μ*m) is presented in Figure 3. Under the compression speed of 0.05 mm/s, the whole deformation and fracture process was different between tantalum and titanium scaffolds. When the internal compressive resistance reached its highest limit (17.9%, 87.6 MPa), the connecting beams among the pores begin to fracture, starting internal collapse. The number of fractured connecting beams increased as compression continued, reducing the stress to minimum at 26.4%, 51.5.4 MPa. The internal material was compacted when the internal space reduced to 0 at 35.4% strain, and the stress restarted to increase until the compression test stopped.

**Figure 4:**
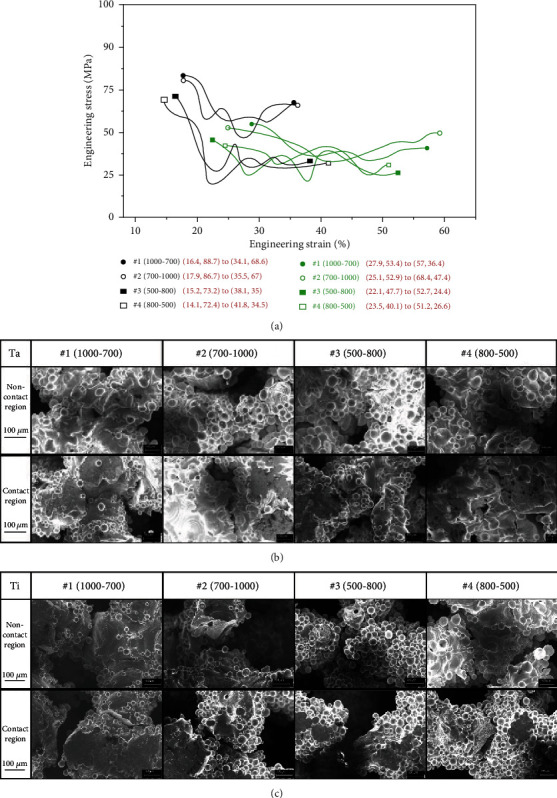
(a) Partial stress-strain curves of all tested scaffolds, which show the process of internal fracture starting to completion. (b) SEM images of 4 kinds of tantalum scaffolds after compression. (c) SEM images of 4 kinds of titanium scaffolds after compression.

Samples were extracted from the eight types of scaffolds at the time of complete internal fracture (Figure 4(a)). There was significant difference between the tantalum and titanium stress/strain curves. Initial point of fracture started at 15% engineering strain in titanium scaffolds, while it was 25% with tantalum scaffolds, indicating higher resistance to deformation of tantalum than titanium, and that tantalum scaffolds can undergo a greater degree of uniform deformation before connecting beam starts fracture. Scanning electron microscope observation showed no obvious microcracks in the contact and noncontact region of all tantalum scaffolds, with width range of microcrack of 14-50 *μ*m (Figure 4(b)). On the other hand, there were obvious microcracks in the contact and noncontact region of all titanium scaffolds, with microcrack width range of 70-210 *μ*m (Figure 4(c)). This difference was consistent with the difference of the two series of stress/strain curves of tantalum and titanium scaffolds (Figure 4(a)).

**Figure 5:**
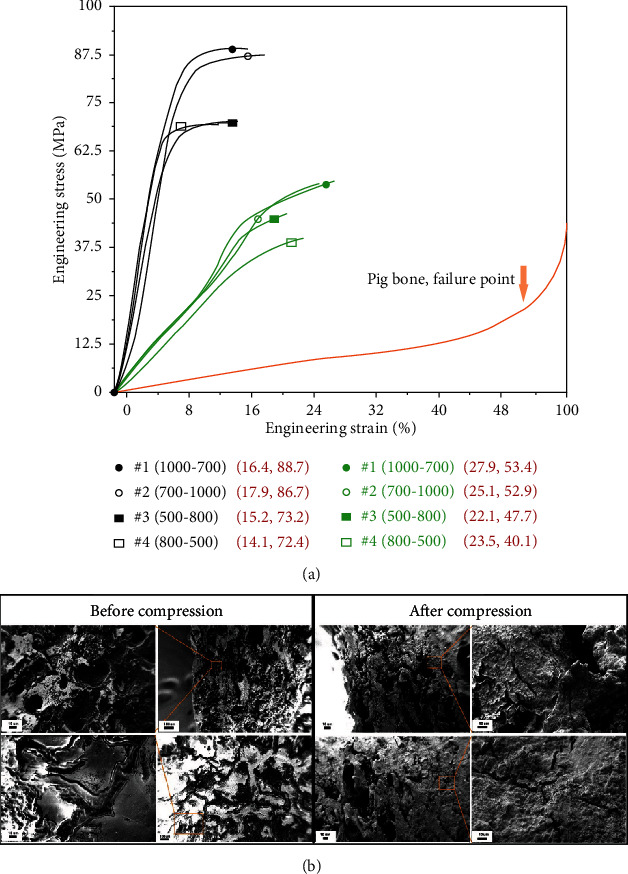
(a) Engineering stress-strain curves of the tantalum and titanium scaffolds before connecting beams start to fracture and the deformation curve of pig bone before failure. (b) SEM images of pig bone sample after compression.

The compression deformation resistance of the above tantalum and titanium scaffolds was compared with that of pig femoral bone; results showed that pig bone scaffolds began compression deformation about 50% before the internal bone beams began to fail, which was much later than that of tantalum and titanium (Figure 5(a)). And the SEM images in Figure 5(b) showed that the width of cracks in pig femoral bone scaffold was significantly smaller than 10 *μ*m. When compared with titanium scaffolds, the deformation behavior and stress-strain parameters of tantalum scaffolds are closer to that of pig bone scaffolds (0.61 ± 0.07 GPa-0.83 ± 0.09 GPa).

## 4. Discussion

Artificial biocompatible implants are needed in various orthopedic surgeries such as joint replacement surgeries, orthopedic reconstruction of the bone defects due to tumor, infection, and trauma as well as congenital deformities. Ideal implants in those occasions are those with fine biocompatibility, osteogenic induction capability, and bone like biomechanical properties.

Titanium alloys are the most commonly used materials for orthopedic implants. Laser engineering technique was used to fabricate low-modulus, tailored porous titanium alloy structure. The titanium alloy with 23-32% porosity is similar to that of cortical bone, and a 16-week study on rats showed fine integration between the implant and bone tissue. It is reported that, by laser engineered net shaping technique, it is possible to construct complex 3-dimentional titanium structures and found that titanium structure with 35-42 vol.% porosity is similar to that of human cortical bone. In a previous study, titanium implants with porosity of 17-58% and pore size of 800 *μ*m were fabricated using laser engineered net shaping method. They showed excellent mechanical strength, strong cell adhesion, and more extracellular matrix when experimented with human osteoblast cells.

However, due to bioinert surface, titanium alloys display poor biological response in vivo. This might be overcome with various surface modification techniques such as coatings with more biocompatible materials. Previous studies used rat and rabbit models to prove the possibility of improving titanium biocompatibility by 3D printed tantalum coatings and found that microporosity design and nanoscale surface modification significantly increased the cytocompatibility and osseointegration of the implant.

Although titanium implants are widely used in various orthopedic, spinal, and dental procedures, there are reports that tantalum scaffolds are superior to titanium in terms of bone induction and osseointegration. By directly comparing additively manufactured porous titanium and tantalum implants for their osseointegration properties using rat distal femur model for five and 12 weeks, previous studies found that there are no significant differences between titanium and tantalum implant in terms of osteointegration 5 weeks after surgery. However, porous tantalum scaffolds showed higher osteoid formation at 12 weeks after surgery, indicating better osseo-inductive properties of tantalum than titanium. Lu et al. [25] used bone marrow mesenchymal stem cells from ovariectomized rats to study cellular activity on tantalum and titanium plates and found that tantalum can better promote cell adhesion, proliferation, and osteogenic differentiation than titanium plates. When used to bridge the femoral bone defect of ovariectomized rats, the amount of new bone formation on the surface of tantalum plate was significantly larger than that of titanium plate. Further studies showed that the genetic expression and protein secretion of osteocalcin, type I collagen, and the formation of calcium nodules were significantly higher on the surface of tantalum than that of titanium when cocultured with bone marrow mesenchymal stem cells. Based on the gene expression of integrin *α*5, *β*1, and extracellular signal regulated kinase (Erkl/2) on the surface of tantalum plates, it was speculated that tantalum may have higher osteogenic induction properties through integrin *α*5*β*1/Erkl/2 signaling pathway [26]. Shi et al. [27] also found that the osteogenic differentiation on the surface of tantalum plates was better than titanium, but they believed that tantalum-mediated osteogenic differentiation was achieved through Wnt/*β*-catenin and TGF-*β*/smad signaling pathways. In our previous studies, we have also found that 3D printed tantalum implants can provide excellent structural support for patients with large iliac tumors and long femoral bone defect due to postoperative infection [28, 29]. However, there are few studies comparing the biomechanical properties of 3D printed tantalum and titanium scaffolds with different diameters.

In order to further evaluate the biomechanical properties of tantalum and titanium scaffolds to reconstruct bone defect, here we tested 3D printed tantalum and titanium scaffolds with different pore diameters. Results of our study showed that the equivalent stress of four types of scaffolds was all significantly larger in tantalum scaffolds than titanium scaffolds, while the range of Young's modulus of tantalum was significantly lower than titanium scaffolds. In a separate series of studies, we found that the pore diameter of 1000-700 *μ*m is more suitable for tantalum scaffolds to induce osseointegration, while 700-1000 *μ*m is more suitable for titanium (unpublished data). The engineering stress-strain curves showed that tantalum scaffolds can undergo a greater degree of uniform deformation before connecting beams start to fracture, which was later proved by scanning electron microscope tests. Further analysis on same size pig femoral bone scaffolds showed that the deformation behavior and stress-strain parameters of tantalum scaffolds are closer to that of pig bone scaffolds than titanium scaffolds. In in vitro studies, microcracks were formed because the compression strength exceeded the bearing strength of the scaffolds. The porous implants themselves are strong enough to be used as implants, and their strength is further increased with osseous integration 4-6 weeks after surgery.

Other metals such as magnesium and zinc have great potential as bone substitutes. However, their application is limited due to unfavorable biomechanical properties. Yang et al. used laser additive manufacturing technique to use graphene oxide reinforcement in zinc scaffold, which simultaneously enhanced the strength and ductility of zinc scaffold. They contributed the enhanced strength to the grain refinement and orientation, efficient load shift, and the Orowan strengthening by the homogeneously distributed graphene oxide reinforcement [30]. Yang et al. also used sol-gel method to synthesize mesoporous bioglass and used laser additive manufacturing to infuse the mesoporous bioglass into Mg-based composite, which showed significantly increased osseointegration and enhanced corrosion resistance [31]. In our study, we directly compared the 3D printed tantalum and titanium scaffolds and found that tantalum had better biomechanical characteristics than the titanium scaffolds. Further mechanical tests can be carried out to compare the mechanical characteristics of tantalum as compared with other metals such as Mg and Zn except for titanium in the future studies.

It is clear from the current study that tantalum scaffolds are superior to titanium scaffolds in stress-strain curves and more similar to animal bones. Further osseous integration experiments are still needed to further validate the potential of tantalum scaffolds as replacement for titanium scaffolds.

## 5. Conclusion

3D printed tantalum scaffolds are superior to titanium scaffolds in resistance to compression and deformation and have biomechanical properties closer to bone scaffolds.

## Figures and Tables

**Table 1:** Equivalent stress of four types of scaffolds.

Diameter	Tantalum	Titanium	*P*
1000-700 *μ*m	403 ± 1.51 MPa	201 ± 4.61	<0.01
700-1000 *μ*m	411 ± 1.43 MPa	212 ± 1.73	<0.01
500-800 *μ*m	389 ± 1.84 MPa	214 ± 3.81	<0.01
800-500 *μ*m	404 ± 1.69 MPa	191 ± 2.14	<0.01

## Data Availability

The original data is available from the corresponding author upon request.
