# Correction to “Highly Porous 3D Printed Tantalum Scaffolds Have Better Biomechanical and Microstructural Properties Than Titanium Scaffolds”

**DOI:** 10.1155/bmri/9793730

**Published:** 2025-12-30

H. Fan, S. Deng, W. Tang, et al., “Highly Porous 3D Printed Tantalum Scaffolds Have Better Biomechanical and Microstructural Properties Than Titanium Scaffolds,” *BioMed Research International*, 2021, (2021): 2899043, https://doi.org/10.1155/2021/2899043.

In the article, the authors have identified that, in Figures 1 and 5, the incorrect panels were selected during the figure preparation prior to submission.

After assessment from the editorial board of the author′s response, the errors do not affect the results or conclusions of the article. The correct Figures 1 and 5 are shown as follows:

Figure 1(a) Four kinds of 3D modeling scaffolds and the corresponding biological sites. (b) SEM images of four kinds of tantalum scaffolds before compression. (c) SEM images of four kinds of titanium scaffolds before compression.

**Figure:**
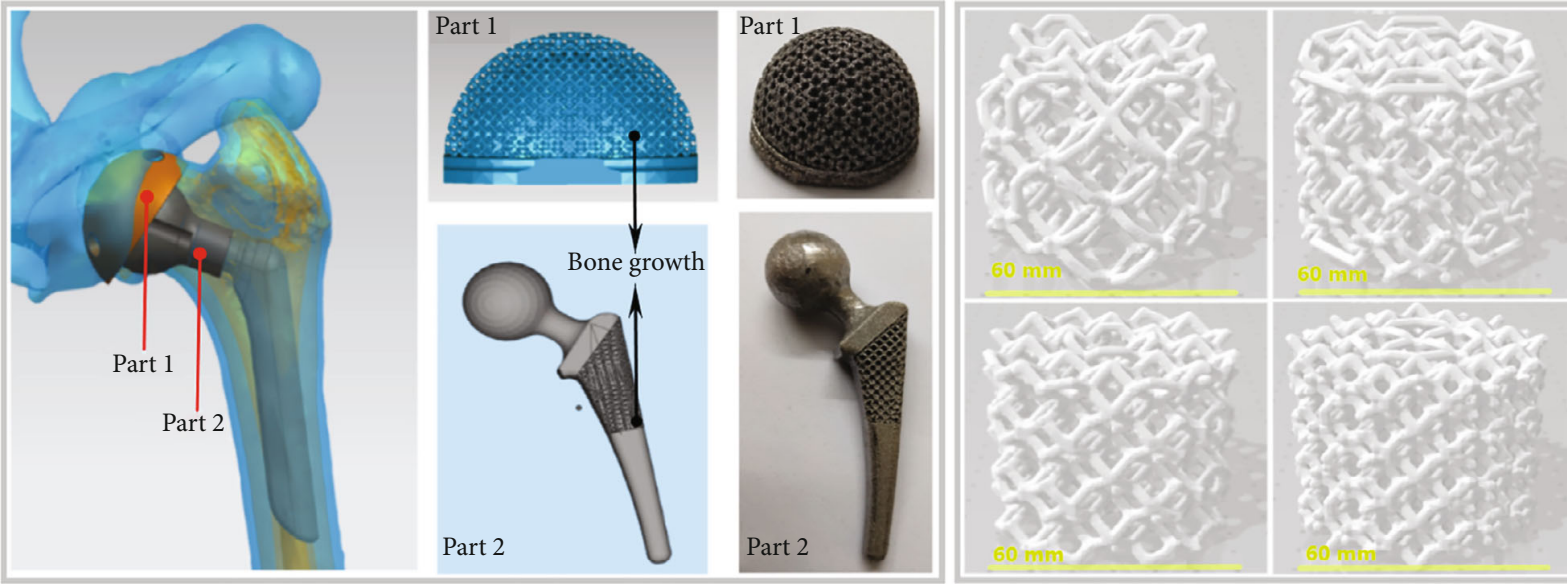
(a)

**Figure:**
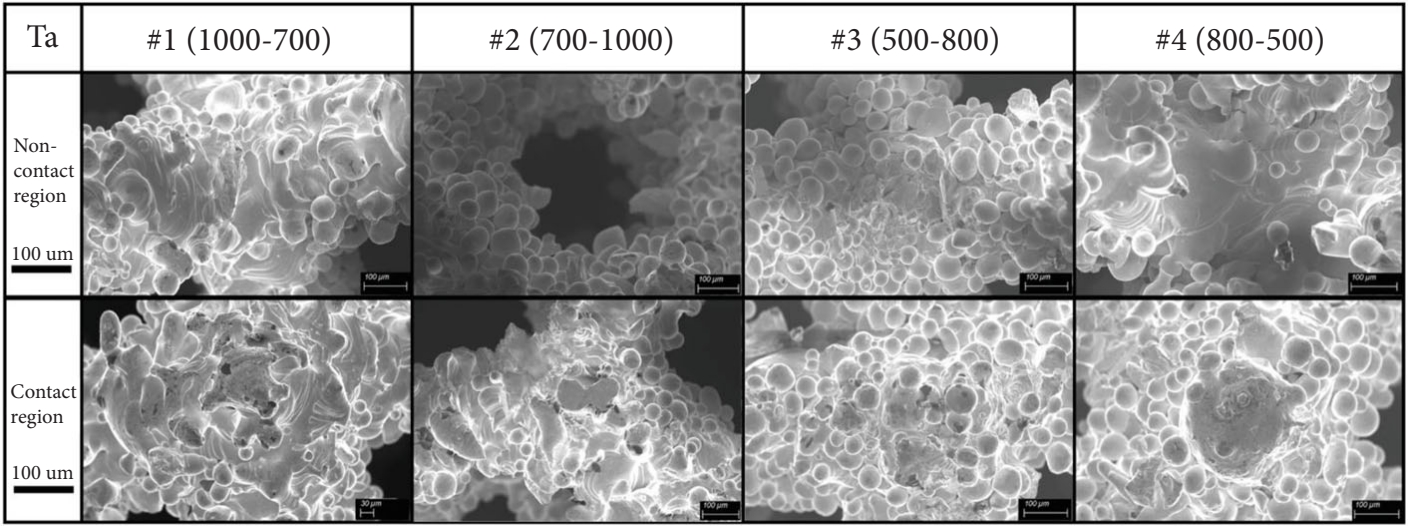
(b)

**Figure:**
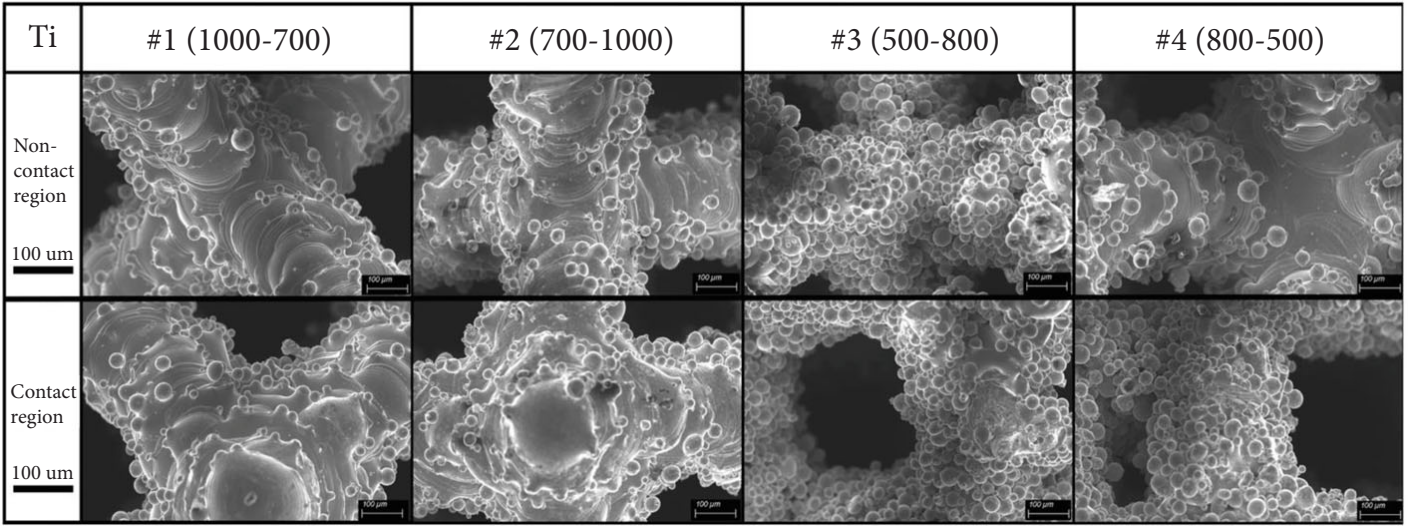
(c)

Figure 5(a) Engineering stress–strain curves of the tantalum and titanium scaffolds before connecting beams start to fracture and the deformation curve of pig bone before failure. (b) SEM images of the pig bone sample after compression.

**Figure:**
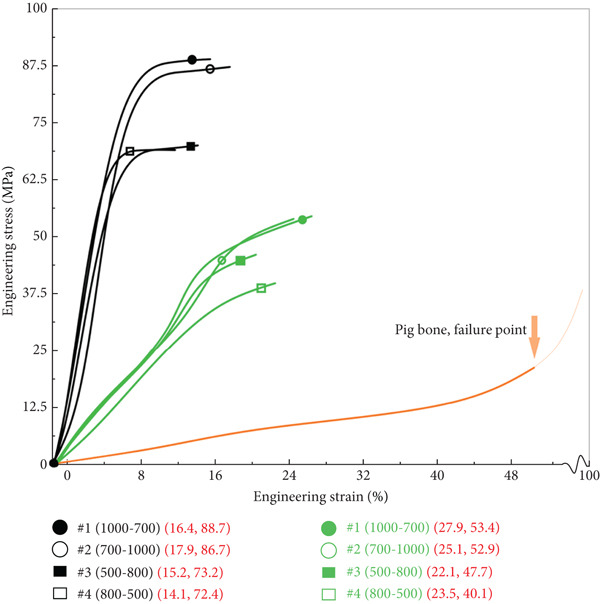
(a)

**Figure:**
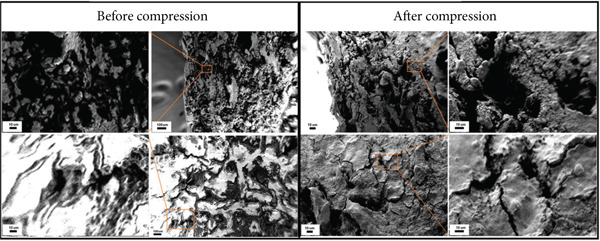
(b)

We apologize for these errors.

